# An epidemic of cataract surgery in Korea: the effects of private health insurance on the National Health Insurance Service

**DOI:** 10.4178/epih.e2024015

**Published:** 2024-01-06

**Authors:** Hyejin Lee, Soo-Hee Hwang, Choon-Seon Park, Seol-Hee Chung, Catherine L. Chen, Jin Yong Lee, Jin Soo Lee

**Affiliations:** 1 Department of Family Medicine, Seoul National University Bundang Hospital, Seongnam, Korea; 2 Department of Family Medicine, Seoul National University, Seoul, Korea; 3 HIRA Research Institute, Health Insurance Review & Assessment Service, Wonju, Korea; 4 Department of Anesthesia & Perioperative Care, Philip R. Lee Institute for Health Policy Studies, University of California, San Francisco, CA, USA; 5 Public Healthcare Center, Seoul National University Hospital, Seoul, Korea; 6 Department of Health Policy and Management, Seoul National University College of Medicine, Seoul, Korea; 7 Institute of Health Policy and Management, Seoul National University Medical Research Center, Seoul, Korea; 8 Health Insurance Review & Assessment Service, Wonju, Korea

**Keywords:** Cataract surgery, National Health Insurance, Private health insurance, Medical cost, Excess surgery

## Abstract

**OBJECTIVES:**

In Korea, the National Health Insurance Service (NHIS) covers essential healthcare expenses, including cataract surgery. To address concerns that private health insurance (PHI) might have inflated the need for such procedures, we investigated the extent of the PHI-attributable increase in cataract surgery and its impact on NHIS-reimbursed expenses.

**METHODS:**

This retrospective, observational study uses nationwide claims data for cataract surgery from 2016 to 2020. We examined trends in utilization and cost, and we estimated the excess numbers of (1) cataract operations attributable to PHI and (2) types of intraocular lenses used for cataract surgery in 2020.

**RESULTS:**

Between 2016 and 2020, a 36.8% increase occurred in the number of cataract operations, with increases of 63.5% and 731.8% in the total healthcare costs reimbursed by NHIS and PHI, respectively. Over a 5-year period, the surgical rate per 100,000 people doubled for patients aged <65 years (from 328 in 2016 to 664 in 2020). Among the 619,771 cases in 2020 of cataract surgery reimbursed by the Korean diagnosis-related group system, more non-NHIS-covered intraocular lenses were used for patients aged <65 years than ≥65 years (68.1 vs. 14.2%). In 2020 alone, an estimated 129,311 excess operations occurred, accounting for an excess cost of US$115 million.

**CONCLUSIONS:**

A dramatic increase in the number and cost of cataract operations has occurred over the last 5 years. The PHI-related increase in operations resulted in increased costs to NHIS. Measures to curtail the non-indicated use of cataract surgery should be implemented regarding PHI.

## GRAPHICAL ABSTRACT

[Fig f5-epih-46-e2024015]

## Key Message

In Korea, the National Health Insurance Service (NHIS) covers essential healthcare expenses including cataract surgery. There are concerns that private health insurance (PHI) might have boosted cataract surgery. Over 5 years, there was a 36.8% increase in the number of cataract surgery with 63.5% and 731.8% increase in total healthcare costs reimbursed by NHIS and PHI, respectively. Cataract surgeries predominantly surged among individuals under the age of 65, accompanied by a greater utilization of non-NHIS-covered intraocular lenses. The estimated excess number of surgeries was 129,311 in 2020 alone, which accounted for an excess cost of $115 million.

## INTRODUCTION

Cataracts are the most common cause of visual impairment among older adults [1,2]. Ophthalmic surveys of 2008-2012, which examined 19,953 adults aged ≥ 40 years with a slit lamp, reported a 42% overall prevalence of cataracts, which increased rapidly from 11.1% among individuals aged 40-49 years to 35.7%, 71.8%, and 94.2% for individuals aged 50-59 years, 60-69 years, and ≥ 70 years, respectively [3]. Cataract surgery is the most effective means of restoring vision [3,4], and 8-53% of patients with cataracts are reported to undergo surgery [5,6]. Initially, cataract surgery was performed to prevent blindness; however, surgery is also recommended to improve quality of life and prevent injuries that may arise from decreased visual acuity [4,7,8]. Therefore, the decision to proceed with cataract surgery is typically made jointly by the patient and ophthalmologist when the presence of cataract disease begins to compromise the patient’s quality of life [4,9]. The healthcare system, including a patient’s insurance policy regarding the scope of coverage and copayment levels, also affects the patient’s decision regarding whether to undergo cataract surgery considering the out-of-pocket expenses.

The Korean healthcare system is based on compulsory social health insurance, and the National Health Insurance Service (NHIS) is the sole insurer that has provided mandatory universal health coverage (UHC) to all Korean residents since 2000; 97% of the subscribers are NHIS beneficiaries paying an insurance premium, and 3% are recipients of the government’s Medical Aid (MA) program [10]. Generally, the out-of-pocket copayment or coinsurance rate is 20-30% [11]. The basic payment scheme is a fee-for-service model. Some diseases and procedures are reimbursed by the diagnosis-related group (DRG) payment system, including cataract surgery since 2013. Subsequently, a steady increase in the number of cataract operations has occurred [12], and recently, PHI claims for cataract surgery have surged [13].

PHI is classified into primary, duplicate, complementary, and supplementary health insurance based on its role and relationship within the healthcare system. In Korea, PHI primarily serves the functions of supplementary and complementary health insurance [14]. Currently, approximately 34 million people (67% of the Korean population) are enrolled in PHI [12]. Since the inception of PHI in 2003, to expand market share, PHI companies have competitively marketed insurance products with exceptional terms and conditions that fully compensate healthcare expenses not covered or only partially covered by the NHIS.

Healthcare utilization varies according to changes in the cost-sharing method. Previous studies have reported that as the patient’s out-of-pocket costs increased, their usage of medical treatment and medication decreased [15-17]. PHI, which reduces out-of-pocket spending, is known to lead to increased healthcare utilization and greater inequality [18]. An Australian study reported similarly that PHI’s reduction in out-of-pocket costs led to increased healthcare utilization [19].

In Korea, since the cost of expensive premium intraocular lenses (IOLs) is covered by PHI while the cost of cataract surgery itself is reimbursed by NHIS, major concerns were raised that PHI might affect healthcare utilization by removing the cost-sharing mechanism, which is negatively correlated with healthcare utilization [20]. Another concern is that refractive lens exchange surgery, which is not covered by NHIS, could be claimed as cataract surgery for reimbursement purposes. In this study, we sought to investigate the extent to which PHI increased patients’ healthcare utilization and the expenses of cataract surgery reimbursed by NHIS.

## MATERIALS AND METHODS

### Study oversight and data sources

Using the Health Insurance Review and Assessment Service (HIRA) claims database, which covers all NHIS beneficiaries in Korea (including MA recipients, whose healthcare expenses are paid by the government), we extracted the demographic characteristics and comorbidities of patients who underwent cataract surgery as well as information about the medical facilities where the procedures were performed. Since the total reimbursement amount for cataract surgery covered by supplemental PHI was disclosed beginning in 2016, we used claims data filed between January 1, 2016 and December 31, 2020.

Since 2013, the DRG payment system has been required in all medical institutions that perform cataract surgery. We extracted all inpatient claims data regarding cataract surgery using Korean DRG codes C051, C052, C053, and C054 (cataract surgery) and Korean Disease codes H25, H26, H28, and Q12.0 (cataract). We also used data regarding the total reimbursement by supplemental PHI for cataract operations between 2016 and 2020 from the Korea Insurance Research Institute [13], as well as demographic data regarding resident registration by age group from the Korea Ministry of the Interior and Safety (MOIS) [21]. Places of surgery were classified into tertiary hospitals, general hospitals, hospitals, and clinics, according to the institution’s size and number of beds. Except for the MOIS data, which were used for sex and age distribution values, all data were considered at the individual level.

### The rates and medical costs of cataract surgery

We defined the rate of cataract surgery as the number of cataract operations performed annually per 100,000 people [22]. Premium lenses are covered only by PHI contracts signed before January 2016, whereas they are not covered by the NHIS, according to its reimbursement regulations. The total medical cost (α) was defined as the sum of costs covered by the NHIS (β^i^) and PHI (γ^i^). The NHIS-covered cost (β^i^) was calculated as the sum of the NHIS-reimbursed cost (β_A_^i^) and coinsurance (β_B_^i^). The NHIS-reimbursed cost (β_A_^i^) was calculated as the difference between the DRG billing cost (β_AD_^i^) and the lens cost for patients with PHI (β_AL_^i^).

### Total medical cost (α)


$$=\sum_{i=1}^{n}\left\{\left[\left(\mathrm{DRG}\;\mathrm{billing}\;\mathrm{cost}\;(\beta_{AD}^{i})-\mathrm{lens}\;\mathrm{cost}\;\mathrm{for}\;\mathrm{patients}\;\mathrm{with}\;\mathrm{PHI}\;(\beta_{AL}^{i})\right)+\mathrm{coinsurance}(\beta_{B}^{i})\right]+\mathrm{PHI}\;\mathrm{covered}\;\mathrm{cost}\;(\gamma^{i})\right\}$$

We used the 2016 rate for cataract surgery as a baseline reference for estimating the excess number of operations and related medical costs. All costs were converted to US dollars from Korean won (exchange rate of 1,176.93:1.00, as of December 1, 2021).

### Estimating the excess number of cataract operations

Population aging has a significant impact on the rate at which cataract operations may occur. Therefore, when estimating the excess number of operations, we had to consider the rapid aging of the Korean population. We assumed that the rates of surgery by sex and age group in 2016 would be maintained in subsequent years; the expected number of operations after 2016 was estimated by standardizing the sex and age groups at 5-year intervals using the 2016 sex-specific and age-specific rates as a baseline reference. Subsequently, we calculated the differences between the observed and expected numbers of operations for each sex and age group. A negative difference indicated no occurrence of excess operations; therefore, we did not include negative values in the analysis.

In a reform of the DRG reimbursement policy, a new rule was implemented as of January 1, 2020, such that the cost of IOLs would not be reimbursed by the DRG payment system if the surgery was performed without NHIS-covered IOLs. Since a specific code was recorded to indicate a non-NHIS-covered IOL within the DRG system, we also analyzed the characteristics of cataract operations based on the types of IOL used for patients having cataract surgery in 2020.

### Statistical analysis

Baseline characteristics are presented as mean and standard deviation (SD) or number and percentage, using the cataract surgical procedure (rather than the individual patient) as the unit of analysis unless otherwise stated. Since cataract surgery is common among older adults, the age-based data were divided into two large subgroups (< 65 and ≥ 65 years) as well as smaller groups at 5-year intervals. To elucidate temporal trends, we used the Korean resident population data of the MOIS between 2016 and 2020 to calculate age-group-specific surgical rates per 100,000 people [13]. Differences in categorical and continuous variables were assessed using the chi-square test and the student t-test, respectively. All statistical analyses were performed using SAS version 9.4 (SAS Institute Inc., Cary, NC, USA).

### Ethics statement

This study was reviewed and approved by the Institutional Review Board of the HIRA (IRB No. 2021126-001).

## RESULTS

### Characteristics of cataract surgery

The number of cataract operations increased by 36.8% from 475,568 in 2016 to 650,355 in 2020, with a mean annual increase of 8.3%. The number of patients who underwent surgery increased by 26.7% from 327,476 in 2016 to 415,072 in 2020. The mean± SD age of patients decreased from 69.0± 10.2 years in 2016 to 65.9± 10.3 years in 2020. In the group aged < 65 years, a gradual increase was found in the number and proportion of cataract operations, from 146,789 (30.9%) in 2016 to 287,689 (44.2%) in 2020; however, no change was observed in the group aged ≥ 65 years. More female than male patients underwent surgery (60.2 vs. 39.8% in 2020). Most patients were covered by the NHIS, and only a small percentage were MA recipients (0.2% in 2016 and 0.4% in 2020). Regarding the type of institution where cataract surgery was performed, the percentage of operations at a clinic increased in 2020 from 77.5% to 80.9%, whereas the percentage at tertiary hospitals decreased from 6.9% in 2016 to 4.7% in 2020, respectively (Table 1).

### Trends in rates of surgery per 100,000 people, stratified by age group

Overall, the rate of cataract surgery was much higher in the group aged ≥ 65 years than in the group aged < 65 years (4,269 and 664 operations per 100,000 people, respectively, in 2020). In the older group, no drastic changes in rate were found, with a decreasing trend over the 5-year period (from 4,700/100,000 people in 2016 to 4,269/100,000 people in 2020), except for a small increase to 4,939/100,000 people in 2019. In contrast, the rate of surgery in the group aged < 65 years doubled from 328/100,000 people in 2016 to 664/100,000 people in 2020 (Supplementary Material 1).

**Figure 1.**
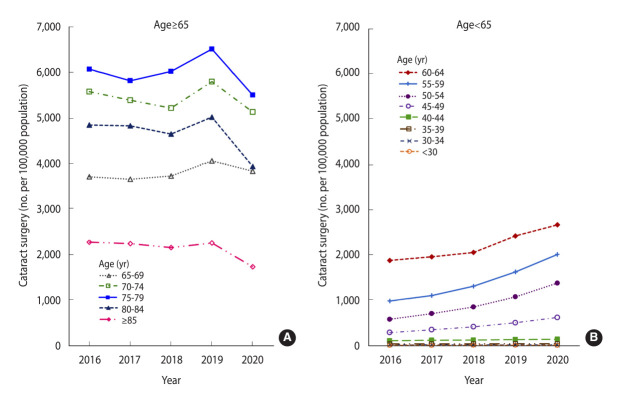
Rates of cataract surgery by age group (A) ≥65 and (B) <65 from 2016 to 2020.

Regarding analysis by 5-year intervals, the group aged 75-79 years showed the highest rates of cataract surgery, followed by the groups aged 70-74 years, 80-84 years, and 65-69 years, respectively. Notably, the rates of surgery decreased in 2020 compared to those in 2019 for all age subgroups ≥ 65 years (Figure 1A, Supplementary Material 1). This could be attributed to the effects of the coronavirus disease 2019 pandemic. In contrast, the rate of surgery in the < 65 years age group increased from 548/100,000 people in 2019 to 664/100,000 people in 2020. Over the 5-year study period, a continuous increase was observed in the rates of cataract surgery in the subgroups aged 45-49 years, 50-54 years, 55-59 years, and 60-64 years, by 113.7%, 137.4%, 103.8%, and 41.8%, respectively (Figure 1B, Supplementary Material 1).

### Trends in the medical costs of cataract surgery

**Figure 2.**
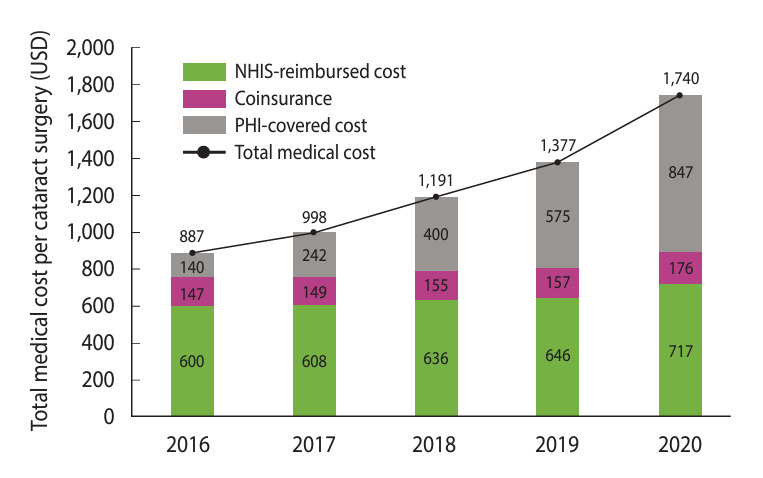
Average medical costs for cataract surgery from 2016 to 2020. NHIS, National Health Insurance Service; PHI, private health insurance; USD, US dollar.

The total medical costs of cataract surgery increased by 168% from US$421.1 million in 2016 to US$1.1 billion in 2020. Specifically, the NHIS-covered costs (NHIS-reimbursed costs and coinsurance) gradually increased by 63.5% from US$354.9 million in 2016 to US$580.2 million in 2020. Within the same period, the PHI-covered cost showed a sharper increase of 731.8%, from US$66.2 million to US$550.8 million. In 2020, PHI covered 48.7% of the total medical costs of cataract operations (US$1,130.9 billion) (Figure 2, Supplementary Material 2). The medical cost of each cataract procedure, which was calculated as the simple average per procedure, increased by 96.1% from US$887 in 2016 to US$1,740 in 2020. Although some of the statutory out-of-pocket costs may have overlapped with the PHI-covered costs, the overlap was considered insignificant (Supplementary Material 2).

### Estimated excess number of cataract operations

**Figure 3.**
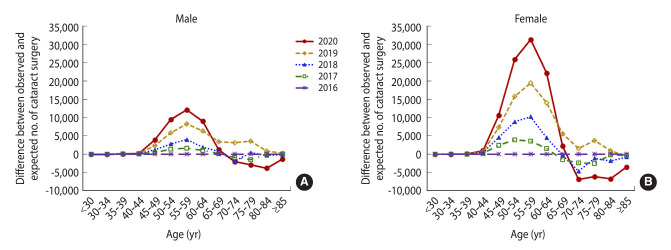
Differences between observed and expected numbers of cataract operations, by 5-year intervals (A) male and (B) female.

In 2020, the observed number of cataract operations exceeded the expected number by 129,311 (Supplementary Material 3). Further analysis according to sex revealed that female patients had 2.0 times to 2.6 times more excess operations than male patients (Figure 3A and B, Supplementary Material 4).

### Types of intraocular lenses used for cataract surgery in 2020

Among the 650,355 cataract operations performed in 2020, 619,771 (95%) were covered by the DRG payment system. Among the DRG-reimbursed operations, 38.3% were performed using non-NHIS-covered IOLs. Overall, the mean age of patients who underwent cataract surgery using non-NHIS-covered IOLs was 12 years younger than the patients who used NHIS-covered IOLs (mean age: 58.38± 7.88 vs. 70.47± 8.84 years; p< 0.001).

Non-NHIS-covered IOLs were used in 68.1% and 14.2% of cataract operations performed in the groups aged < 65 years and ≥ 65 years, respectively. The percentage of operations performed using non-NHIS-covered IOLs was 10.2% higher for female patients (42.3%) than male patients (32.1%). Additionally, the percentages of cataract operations performed using non-NHIS-covered IOLs were significantly lower in tertiary (11.3%) and general hospitals (9.1%) than in hospitals (37.3%) and clinics (40.9%). Overall, 90.6% of the cataract operations that used non-NHIS-covered IOLs were performed in clinics.

**Figure 4.**
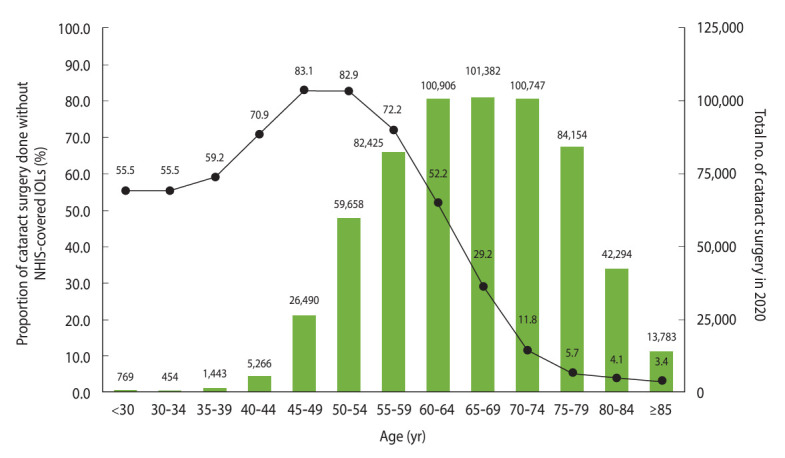
Percentages of cataract operations using premium IOLs and total numbers of cataract operations, by 5-year intervals. Premium IOLs are not covered by NHIS. Results were stratified by patient age in 5-year intervals. This figure only includes cataract operations reimbursed by the diagnosis-related group payment system in 2020. NHIS, National Health Insurance Service; IOLs, intraocular lenses.

For the subgroups aged <65 years, non-NHIS-covered IOLs were used for 83.1%, 82.9%, 72.2%, and 52.2% of the operations performed for the groups aged 45-49 years, 50-54 years, 55-59 years, and 60-64 years, respectively. For the subgroups aged ≥ 65 years, a gradual decrease occurred in the percentage of cataract operations performed using non-NHIS-covered IOLs, from 29.2% in the subgroup aged 65-69 years to 3.4% in the subgroup aged ≥ 85 years. Analysis by sex revealed that the percentage of operations performed using NHIS-covered IOLs increased with age, starting from the subgroups aged 60-64 years for males and 65-69 years for females (Figure 4, Supplementary Materials 5 and 6).

## DISCUSSION

Our findings showed that the overall rates of cataract surgery in Korea increased between 2016 and 2020, with substantial increases in rates for patients younger than 65 years. Furthermore, an incremental increase occurred in the excess number of cataract operations, primarily in patients younger than 65, with females showing a higher excess number of operations than males. This trend was most notable in the year 2020 when, because of the severe acute respiratory syndrome coronavirus 2 pandemic, the rate of cataract surgery declined compared to 2019 and the observed number of operations in patients older than 65 years fell below the expected total, in contrast to the excess number of operations observed the same year in patients younger than 65 years. Taken together, our findings suggest that factors other than an aging population have contributed to the increased utilization of cataract surgery in Korea.

Our findings contradict medical guidelines for cataract care and our epidemiological knowledge of cataract disease [23]. A remote explanation could be overdiagnosis, similar to the thyroid cancer epidemic observed a decade ago in Korea, where widespread screening using very sensitive detection tools, including thyroid ultrasound, resulted in a nationwide overdiagnosis of indolent subclinical thyroid cancers [24]. Although early-stage cataracts may be detected easily through a slit-lamp examination, overdiagnosis cannot sufficiently explain the increased rate of cataract surgery among females aged 45-65 years.

We believe that the most likely driver of increased surgical rates is the use of premium IOLs for vision correction surgery in patients with presbyopia, with claims submitted as “cataract surgery” for NHIS reimbursement purposes. Both the overdiagnosis of thyroid cancer and the overutilization of cataract surgery are likely to be driven by the overutilization of marketed healthcare services covered by the UHC system, which has become a big business in Korea [20,25]. Another contributor to the “cataract surgery epidemic” in Korea is the removal of cost-sharing through the introduction of PHI, since cost-sharing is negatively correlated with healthcare utilization [26]. Since PHI has nearly eliminated the burden of out-of-pocket costs, patients with PHI are incentivized to undergo vision correction surgery using PHI-covered IOLs.

Previous research has shown that risk-averse individuals tend to prefer comprehensive coverage to reduce the substantial financial risk associated with healthcare utilization [27]. Because supplementary PHI reduces out-of-pocket expenses, it may be utilized especially by individuals who seek medical guidance and treatment frequently. Additionally, PHI is known to increase patients’ utilization of services that have relatively elastic demands, rather than emergency services. Cataract surgery is estimated to be directly influenced by the effects of PHI, given its classification under services with relatively elastic demands [18,28]. Healthcare providers working at private clinics widely advertise this surgery because it maximizes the financial benefits of PHI without placing any additional burden on the patient.

Notably, a substantial portion of the NHIS-covered cost of cataract surgery is attributable to the PHI-related increase in the rate of cataract surgery. The actual NHIS-reimbursed cost during the study period was approximately US$893 per surgery. Therefore, the NHIS-reimbursed cost for the 129,311 excess operations was estimated to exceed US$115 million in 2020 alone, which accounted for 24.8% of the NHIS-reimbursed costs of cataract surgery. Even with the assumption that all cataract operations in patients ≥ 65 years and the additional 68.1% of operations performed using non-NHIS-covered IOLs in patients younger than 65 years were indeed medically indicated, an estimated 140,467 cases underwent “cataract surgery” for reasons other than cataracts, which is 11,000 more cases than our estimated excess number of cataract operations. This suggests that our estimate for the excess number of operations is quite conservative and that the actual NHIS-reimbursed costs for the excess number of operations are much greater than our estimate.

Although cataract surgery is generally considered a low-risk procedure [29], it may still result in complications. Intraoperative posterior capsule rupture, intraoperative floppy iris syndrome, and iris or ciliary body injury have been reported in as low as 0.5% to as high as 1.2-5.2% of cataract surgery cases [30], and infections such as endophthalmitis have been reported in 0.04% of the cases [31]. In addition to the immediate complications, since patients who undergo surgery using premium IOLs are relatively young with a longer expected remaining lifespan than typical cataract patients, they may be more likely to experience delayed complications. Given the increase in the number of cataract operations performed in younger patients, these long-term complication rates are expected to increase over time, which will yield additional medical costs to be covered by the NHIS.

Most studies that use insurance claims data have an inherent limitation of distorted coding for reimbursement purposes. Therefore, the current study cannot accurately determine the true prevalence of cataracts; however, this is also a strength of our study [32,33]. By analyzing trends in sex- and age-group-specific rates of cataract surgery, we observed that the recent increase in the surgical rate in Korea was due mostly to the increase in vision correction operations using PHI-covered IOLs in younger patients, which might have been coded as cataract surgery for NHIS reimbursement purposes.

Our study has some additional limitations. In our study, we could not obtain individual-level details on patients who underwent cataract surgery using PHI-covered IOLs. Instead, we only had information regarding the total annual cost of cataract operations covered by PHI. Therefore, the causal effect of PHI on the current cataract surgery epidemic was estimated very conservatively using the sex-group-specific and age-group-specific rates of surgery in 2016 as a baseline. Since PHI for cataract surgery was available starting in 2009, coding distortion may have been present already in the 2016 claims data. Therefore, the actual effect of PHI on the cataract surgery epidemic might be considerably greater than our estimate.

In conclusion, the PHI-related increase in vision correction operations, which were claimed as cataract surgery for NHIS reimbursement purposes, is among the main reasons for the recent increase in the rate of cataract surgery in Korea. This has placed an extra financial burden on the NHIS, which exceeded US$115 million in 2020 alone, accounting for 24.8% of the total NHIS-reimbursed costs of cataract surgery. It is important to establish a clear indication for cataract surgery according to its severity. Moreover, we recommend that reimbursement strategies by PHI and NHIS be revised for cataract surgical care to disallow the non-indicated use of cataract surgery for refractive lens exchange.

## Figures and Tables

**Figure f5-epih-46-e2024015:**
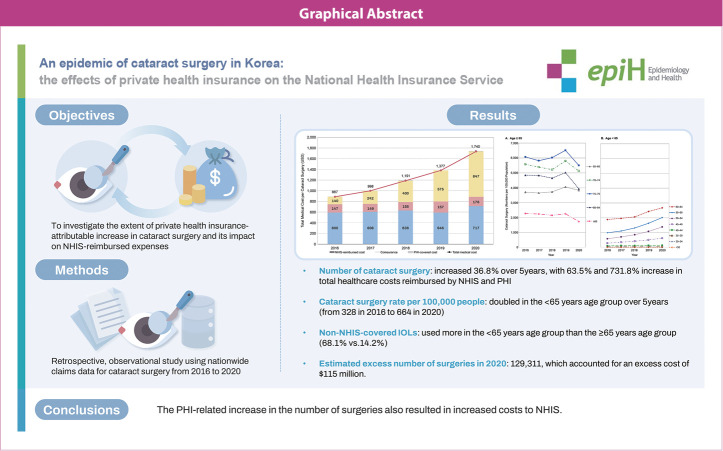


**Table 1.** Characteristics of cataract operations from 2016 to 2020 in Korea

Characteristics	2016	2017	2018	2019	2020
No. of operations	475,568 (100)	503,330 (100)	542,586 (100)	636,628 (100)	650,355 (100)
Age, mean±SD (yr)	69.0±10.2	68.2±10.4	67.7±10.5	67.4±10.4	65.9±10.3
Age distribution (yr)					
<65	146,789 (30.9)	167,063 (33.2)	194,049 (35.8)	240,193 (37.7)	287,689 (44.2)
≥65	328,779 (69.1)	336,267 (66.8)	348,537 (64.2)	396,435 (62.3)	362,666 (55.8)
Sex					
Male	196,432 (41.3)	208,681 (41.5)	223,216 (41.1)	258,034 (40.5)	258,866 (39.8)
Female	279,136 (58.7)	294,649 (58.5)	319,370 (58.9)	378,594 (59.5)	391,489 (60.2)
Type of insurance					
NHIS	474,763 (99.8)	502,540 (99.8)	541,572 (99.8)	634,742 (99.7)	647,811 (99.6)
Medical Aid	805 (0.2)	790 (0.2)	1,014 (0.2)	1,886 (0.3)	2,544 (0.4)
Place of surgery					
Tertiary hospital	32,784 (6.9)	33,828 (6.7)	36,428 (6.7)	38,691 (6.1)	30,756 (4.7)
General hospital	26,513 (5.6)	28,822 (5.7)	30,982 (5.7)	35,413 (5.6)	31,872 (4.9)
Hospital	47,505 (10.0)	54,077 (10.7)	54,476 (10.0)	63,020 (9.9)	61,552 (9.5)
Clinic	368,766 (77.5)	386,603 (76.8)	420,700 (77.5)	499,504 (78.5)	526,175 (80.9)

Values are presented as number (%). SD, standard deviation; NHIS, National Health Insurance Service.

## Supplementary materials

Supplementary Material 1. Trend in cataract surgery rates from 2016 to 2020

Supplementary Material 2. Trend in medical cost of cataract surgery from 2016 to 2020

Supplementary Material 3. Observed and expected number of cataract surgery by 5-year intervals

Supplementary Material 4. Observed and expected number of cataract surgery according to sex by 5-year intervals

Supplementary Material 5. Types of intraocular lens for cataract surgery in 2020

Supplementary Material 6. Types of intraocular lens for cataract surgery in 2020 according to sex and age

## References

[1] Lee CM, Afshari NA (2017). The global state of cataract blindness. Curr Opin Ophthalmol.

[2] West SK, Valmadrid CT (1995). Epidemiology of risk factors for age-related cataract. Surv Ophthalmol.

[3] Prokofyeva E, Wegener A, Zrenner E (2013). Cataract prevalence and prevention in Europe: a literature review. Acta Ophthalmol.

[4] Asbell PA, Dualan I, Mindel J, Brocks D, Ahmad M, Epstein S (2005). Age-related cataract. Lancet.

[5] Park SJ, Lee JH, Kang SW, Hyon JY, Park KH (2016). Cataract and cataract surgery: nationwide prevalence and clinical determinants. J Korean Med Sci.

[6] Laitinen A, Laatikainen L, Härkänen T, Koskinen S, Reunanen A, Aromaa A (2010). Prevalence of major eye diseases and causes of visual impairment in the adult Finnish population: a nationwide population-based survey. Acta Ophthalmol.

[7] Lundström M, Stenevi U (2016). Indications for cataract surgery in a changing world. Acta Ophthalmol.

[8] Chen CL, McLeod SD, Lietman TM, Shen H, Boscardin WJ, Chang HP (2021). Preoperative medical testing and falls in medicare beneficiaries awaiting cataract surgery. Ophthalmology.

[9] National Institute for Health and Care Excellence (2017). Cataracts in adults: management. https://www.nice.org.uk/guidance/ng77.

[10] National Health Insurance Service (2019). 2018 Medical Aid statistics. https://www.hira.or.kr/bbsDummy.do?pgmid=HIRAA020045010000&brdScnBltNo=4&brdBltNo=2318.

[11] Lee H, Lee JR, Jung H, Lee JY (2021). Power of universal health coverage in the era of COVID-19: a nationwide observational study. Lancet Reg Health West Pac.

[12] Organization for Economic Cooperation and Development (OECD) (2020). OECD reviews of public health: Korea: a healthier tomorrow. https://www.oecd.org/health/health-systems/OECD-Reviews-of-Public-Health-Korea-Assessment-and-recommendations.pdf.

[13] Chung SH, Moon HJ (2021). Current status and tasks of private health insurance for cataract surgery. https://www.kiri.or.kr/publication/list.do?docId=31489&catId=29&parentCatId=13&searchCon=SUBJECT&searchWord=National%20Health%20Insurance%20Service.

[14] Choi Y, Kim JH, Yoo KB, Cho KH, Choi JW, Lee TH (2015). The effect of cost-sharing in private health insurance on the utilization of health care services between private insurance purchasers and non-purchasers: a study of the Korean health panel survey (2008-2012). BMC Health Serv Res.

[15] Karaca-Mandic P, Jena AB, Joyce GF, Goldman DP (2012). Out-of-pocket medication costs and use of medications and health care services among children with asthma. JAMA.

[16] Hamina A, Tanskanen A, Tiihonen J, Taipale H (2020). Medication use and health care utilization after a cost-sharing increase in schizophrenia: a nationwide analysis. Med Care.

[17] Hsu J, Price M, Huang J, Brand R, Fung V, Hui R (2006). Unintended consequences of caps on Medicare drug benefits. N Engl J Med.

[18] Ko H (2020). Moral hazard effects of supplemental private health insurance in Korea. Soc Sci Med.

[19] Ludlow T, Fooken J, Rose C, Tang KK (2024). Out-of-pocket expenditure, need, utilisation, and private health insurance in the Australian healthcare system. Int J Health Econ Manag.

[20] Ahn HS, Kim HJ, Welch HG (2014). Korea’s thyroid-cancer “epidemic”--screening and overdiagnosis. N Engl J Med.

[21] Ministry of the Interior and Safety (2022). Population statistics from resident registration. https://jumin.mois.go.kr/index.jsp.

[22] World Health Organization (1997). Global initiative for the elimination of avoidable blindness: an informal consultation. https://www3.paho.org/hq/dmdocuments/2008/Global_Initiative_for_the_Elimination_of_Blindness.pdf.

[23] Liu YC, Wilkins M, Kim T, Malyugin B, Mehta JS (2017). Cataracts. Lancet.

[24] Park S, Oh CM, Cho H, Lee JY, Jung KW, Jun JK (2016). Association between screening and the thyroid cancer “epidemic” in South Korea: evidence from a nationwide study. BMJ.

[25] Ahn HS, Welch HG (2015). South Korea’s thyroid-cancer “epidemic”--turning the tide. N Engl J Med.

[26] Nishi T, Maeda T, Katsuki S, Babazono A (2021). Impact of the 2014 coinsurance rate revision for the elderly on healthcare resource utilization in Japan. Health Econ Rev.

[27] Arrow KJ (2004). Uncertainty and the welfare economics of medical care. 1963. Bull World Health Organ.

[28] Newhouse JP, Price M, McWilliams JM, Hsu J, McGuire TG (2015). How much favorable selection is left in Medicare Advantage?. Am J Health Econ.

[29] Greenberg PB, Liu J, Wu WC, Jiang L, Tseng VL, Scott IU (2010). Predictors of mortality within 90 days of cataract surgery. Ophthalmology.

[30] Moshirfar M, Milner D, Patel BC (2024). Cataract surgery.

[31] Pershing S, Lum F, Hsu S, Kelly S, Chiang MF, Rich WL (2020). Endophthalmitis after cataract surgery in the United States: a report from the intelligent research in sight registry, 2013-2017. Ophthalmology.

[32] Han SJ, Kim KH (2024). Adjusting for confounders in outcome studies using the Korea National Health Insurance Claim Database: a review of methods and applications. J Prev Med Public Health.

[33] Park H, Kim YR, Pyun Y, Joo H, Shin A (2023). Operational definitions of colorectal cancer in the Korean National Health Insurance Database. J Prev Med Public Health.

