# Supplementary material for: An epidemic of cataract surgery in Korea: the effects of private health insurance on the National Health Insurance Service
Source: Epidemiol Health. 2024 Jan 6;46:e2024015. doi: 10.4178/epih.e2024015 (PMC11099570; doi:10.4178/epih.e2024015)
Supplement: Supplementary Material 1. — Trend in cataract surgery rates from 2016 to 2020 [file epih-46-e2024015-Supplementary-1.docx]

Supplementary material 1. Trend in cataract surgery rates from 2016 to 2020

|  | 2016 | | | 2017 | | | 2018 | | | 2019 | | | 2020 | | |
| --- | --- | --- | --- | --- | --- | --- | --- | --- | --- | --- | --- | --- | --- | --- | --- |
| Age group | No. of population | No. of cataract surgery | cataract surgery rate^a^ | No. of population | No. of cataract surgery | cataract surgery rate | No. of population | No. of cataract surgery | cataract surgery rate | No. of population | No. of cataract surgery | cataract surgery rate | No. of population | No. of cataract surgery | cataract surgery rate |
| <30 | 16,805,634 | 1,038 | 6 | 16,550,590 | 994 | 6 | 16,258,188 | 1,035 | 6 | 15,936,280 | 1,012 | 6 | 15,569,559 | 857 | 6 |
| 30-34 | 3,517,868 | 661 | 19 | 3,311,945 | 637 | 19 | 3,189,361 | 571 | 18 | 3,158,230 | 664 | 21 | 3,145,998 | 507 | 16 |
| 35-39 | 4,016,272 | 1,515 | 38 | 4,056,704 | 1,616 | 40 | 4,080,782 | 1,659 | 41 | 3,912,794 | 1,695 | 43 | 3,727,119 | 1,600 | 43 |
| 40-44 | 4,212,243 | 4,702 | 112 | 4,091,743 | 4,987 | 122 | 3,916,194 | 4,906 | 125 | 3,882,192 | 5,190 | 134 | 3,926,316 | 5,542 | 141 |
| 45-49 | 4,584,810 | 13,369 | 292 | 4,611,009 | 16,276 | 353 | 4,572,393 | 19,110 | 418 | 4,501,038 | 22,832 | 507 | 4,368,471 | 27,239 | 624 |
| 50-54 | 4,182,392 | 24,365 | 583 | 4,153,396 | 29,454 | 709 | 4,277,593 | 36,611 | 856 | 4,359,591 | 47,083 | 1,080 | 4,418,728 | 61,148 | 1,384 |
| 55-59 | 4,242,269 | 41,961 | 989 | 4,336,808 | 48,062 | 1,108 | 4,338,291 | 57,099 | 1,316 | 4,307,786 | 70,337 | 1,633 | 4,226,286 | 85,222 | 2,016 |
| 60-64 | 3,139,076 | 59,178 | 1,885 | 3,310,243 | 65,037 | 1,965 | 3,542,849 | 73,058 | 2,062 | 3,765,035 | 91,380 | 2,427 | 3,950,469 | 105,574 | 2,672 |
| 65-69 | 2,237,345 | 82,985 | 3,709 | 2,347,021 | 85,748 | 3,653 | 2,406,790 | 89,704 | 3,727 | 2,545,616 | 103,314 | 4,059 | 2,794,037 | 107,018 | 3,830 |
| 70-74 | 1,781,229 | 99,368 | 5,579 | 1,793,132 | 96,680 | 5,392 | 1,880,192 | 98,082 | 5,217 | 1,972,502 | 114,382 | 5,799 | 2,080,134 | 106,692 | 5,129 |
| 75-79 | 1,457,890 | 88,513 | 6,071 | 1,585,974 | 92,259 | 5,817 | 1,617,744 | 97,415 | 6,022 | 1,623,542 | 105,741 | 6,513 | 1,618,112 | 89,039 | 5,503 |
| 80-84 | 909,130 | 44,042 | 4,844 | 968,146 | 46,747 | 4,829 | 1,032,594 | 47,966 | 4,645 | 1,103,019 | 55,363 | 5,019 | 1,143,935 | 45,024 | 3,936 |
| ≥85 | 610,058 | 13,871 | 2,274 | 661,833 | 14,833 | 2,241 | 713,088 | 15,370 | 2,155 | 782,236 | 17,635 | 2,254 | 859,859 | 14,893 | 1,732 |
| <65 | 44,700,564 | 146,789 | 328 | 44,422,438 | 167,063 | 376 | 44,175,651 | 194,049 | 439 | 43,822,946 | 240,193 | 548 | 43,332,946 | 287,689 | 664 |
| ≥65 | 6,995,652 | 328,779 | 4,700 | 7,356,106 | 336,267 | 4,571 | 7,650,408 | 348,537 | 4,556 | 8,026,915 | 396,435 | 4,939 | 8,496,077 | 362,666 | 4,269 |
| Total | 51,696,216 | 475,568 | 920 | 51,778,544 | 503,330 | 972 | 51,826,059 | 542,586 | 1,047 | 51,849,861 | 636,628 | 1,228 | 51,829,023 | 650,355 | 1,255 |
| Demographic data of resident registration by age group from the Korea Ministry of Interior and Safety were used  ^a^Cataract surgery rate was defined as the number of cataract operations performed per 100,000 people per year. | | | | | | | | | | | | | | | |
