# Supplementary material for: An epidemic of cataract surgery in Korea: the effects of private health insurance on the National Health Insurance Service
Source: Epidemiol Health. 2024 Jan 6;46:e2024015. doi: 10.4178/epih.e2024015 (PMC11099570; doi:10.4178/epih.e2024015)
Supplement: Supplementary Material 2. — Trend in medical cost of cataract surgery from 2016 to 2020 [file epih-46-e2024015-Supplementary-2.docx]

Supplementary material 2. Trend in medical cost of cataract surgery from 2016 to 2020

| Millions of US Dollars^a^ | 2016 | 2017 | 2018 | 2019 | 2020 | Difference 2016-2020 (%) |
| --- | --- | --- | --- | --- | --- | --- |
| Total medical cost (α) | 421.1 | 502.2 | 645.8 | 875.9 | 1,130.9 | 168.6 |
| NHIS covered cost (β) | 354.9 | 380.4 | 428.8 | 510.4 | 580.2 | 63.5 |
| NHIS reimbursed cost (β_A_) | 285.2 | 305.7 | 344.7 | 410.7 | 466.1 | 63.4 |
| Coinsurance (β_B_) | 69. | 74.7 | 84.1 | 99.7 | 114.0 | 63.7 |
| PHI covered cost (γ) ^b^ | 66.2m | 121.7 | 217.0 | 365.5 | 550.8 | 731.8 |
| Medical cost per cataract surgery^c^ (α’) | 887 | 998 | 1,191 | 1,377 | 1,740 | 96.1 |
| NHIS covered cost per cataract surgery (β’) | 747 | 756 | 791 | 802 | 893 | 19.4 |
| NHIS reimbursed cost per cataract surgery (β_A_’) | 600 | 608 | 636 | 646 | 717 | 19.3 |
| Coinsurance per cataract surgery (β_B_’) | 147 | 149 | 155 | 157 | 176 | 19.7 |
| PHI covered cost per cataract surgery (γ’) | 140 | 242 | 400 | 575 | 847 | 505.0 |
| NHIS, National Health Insurance Service; PHI, Private health insurance  ^a^All medical cost calculated in US dollars  ^b^Data on total reimbursement by supplemental PHI for cataract surgery from 2016 to 2020 opened by Korea Insurance Research Institute (2021.7.)  ^c^Cost per cataract surgery was calculated by dividing the total cost by the number of surgeries.  All costs were converted from KRW to USD based on the exchange rate as of December 1, 2021 (1 USD = 1,176.93 KRW). | | | | | | |
