# Supplementary material for: An epidemic of cataract surgery in Korea: the effects of private health insurance on the National Health Insurance Service
Source: Epidemiol Health. 2024 Jan 6;46:e2024015. doi: 10.4178/epih.e2024015 (PMC11099570; doi:10.4178/epih.e2024015)
Supplement: Supplementary Material 3. — Observed and expected number of cataract surgery by 5-year intervals [file epih-46-e2024015-Supplementary-3.docx]

Supplementary material 3. Observed and expected number of cataract surgery by 5-year intervals

|  | 2017 | | | 2018 | | | 2019 | | | 2020 | | |
| --- | --- | --- | --- | --- | --- | --- | --- | --- | --- | --- | --- | --- |
| Age group | Observed | Expected^a^ | Difference | Observed | Expected | Difference | Observed | Expected | Difference | Observed | Expected | Difference |
| <30 | 994 | 1,024 | -30 | 1,035 | 1,005 | 30 | 1,012 | 985 | 27 | 857 | 962 | -105 |
| 30-34 | 637 | 624 | 13 | 571 | 601 | -30 | 664 | 596 | 68 | 507 | 594 | -87 |
| 35-39 | 1,616 | 1,533 | 83 | 1,659 | 1,540 | 119 | 1,695 | 1,478 | 217 | 1,600 | 1,408 | 192 |
| 40-44 | 4,987 | 4,564 | 423 | 4,906 | 4,373 | 533 | 5,190 | 4,332 | 858 | 5,542 | 4,383 | 1,159 |
| 45-49 | 16,276 | 13,453 | 2,823 | 19,110 | 13,340 | 5,770 | 22,832 | 13,134 | 9,698 | 27,239 | 12,744 | 14,495 |
| 50-54 | 29,454 | 24,206 | 5,248 | 36,611 | 24,935 | 11,676 | 47,083 | 25,415 | 21,668 | 61,148 | 25,757 | 35,391 |
| 55-59 | 48,062 | 42,889 | 5,173 | 57,099 | 42,897 | 14,202 | 70,337 | 42,584 | 27,753 | 85,222 | 41,773 | 43,449 |
| 60-64 | 65,037 | 62,376 | 2,661 | 73,058 | 66,727 | 6,331 | 91,380 | 70,927 | 20,453 | 105,574 | 74,400 | 31,174 |
| 65-69 | 85,748 | 87,018 | -1,270 | 89,704 | 89,245 | 459 | 103,314 | 94,338 | 8,976 | 107,018 | 103,567 | 3,451 |
| 70-74 | 96,680 | 99,920 | -3,240 | 98,082 | 104,637 | -6,555 | 114,382 | 109,682 | 4,700 | 106,692 | 115,631 | -8,939 |
| 75-79 | 92,259 | 96,237 | -3,978 | 97,415 | 98,134 | -719 | 105,741 | 98,451 | 7,290 | 89,039 | 98,081 | -9,042 |
| 80-84 | 46,747 | 46,942 | -195 | 47,966 | 50,102 | -2,136 | 55,363 | 53,561 | 1,802 | 45,024 | 55,583 | -10,559 |
| ≥85 | 14,833 | 15,076 | -243 | 15,370 | 16,284 | -914 | 17,635 | 17,912 | -277 | 14,893 | 19,745 | -4,852 |
| Total | 503,330 | 495,862 | 7,468 | 542,586 | 513,820 | 28,766 | 636,628 | 533,395 | 103,233 | 650,355 | 554,628 | 95,727 |
| Excess^b^ |  |  | 16,424 |  |  | 39,120 |  |  | 103,510 |  |  | 129,311 |
| ^a^Expected number of cataract surgery was calculated on the assumption that the age group- and gender-specific cataract surgery rate in 2016 was maintained in subsequent years. | | | | | | | | | | | | |
| ^b^Excess number of cataract surgeries was calculated by adding up only those of exceeding the expected number of surgeries by age group. | | | | | | | | | | | | |
