# Supplementary material for: An epidemic of cataract surgery in Korea: the effects of private health insurance on the National Health Insurance Service
Source: Epidemiol Health. 2024 Jan 6;46:e2024015. doi: 10.4178/epih.e2024015 (PMC11099570; doi:10.4178/epih.e2024015)
Supplement: Supplementary Material 4. — Observed and expected number of cataract surgery according to sex by 5-year intervals [file epih-46-e2024015-Supplementary-4.docx]

Supplementary material 4. Observed and expected number of cataract surgery according to sex by 5-year intervals

|  | 2017 | | | | | | | | |  |
| --- | --- | --- | --- | --- | --- | --- | --- | --- | --- | --- |
|  | Total | | | Male | | | Female | | |  |
| Age group | Observed | Expected^a^ | Difference | Observed | Expected | Difference | Observed | Expected | Difference |  |
| <30 | 994 | 1,024 | -30 | 656 | 636 | 20 | 338 | 388 | -50 |  |
| 30-34 | 637 | 624 | 13 | 406 | 403 | 3 | 231 | 221 | 10 |  |
| 35-39 | 1,616 | 1,533 | 83 | 1,049 | 992 | 57 | 567 | 541 | 26 |  |
| 40-44 | 4,987 | 4,564 | 423 | 2,975 | 2,831 | 144 | 2,012 | 1,733 | 279 |  |
| 45-49 | 16,276 | 13,453 | 2,823 | 7,867 | 7,439 | 428 | 8,409 | 6,014 | 2,395 |  |
| 50-54 | 29,454 | 24,206 | 5,248 | 13,098 | 11,745 | 1,353 | 16,356 | 12,461 | 3,895 |  |
| 55-59 | 48,062 | 42,889 | 5,173 | 21,126 | 19,483 | 1,643 | 26,936 | 23,406 | 3,530 |  |
| 60-64 | 65,037 | 62,376 | 2,661 | 27,505 | 26,444 | 1,061 | 37,532 | 35,932 | 1,600 |  |
| 65-69 | 85,748 | 87,018 | -1,270 | 35,565 | 35,356 | 209 | 50,183 | 51,662 | -1,479 |  |
| 70-74 | 96,680 | 99,920 | -3,240 | 38,541 | 39,396 | -855 | 58,139 | 60,524 | -2,385 |  |
| 75-79 | 92,259 | 96,237 | -3,978 | 36,380 | 37,832 | -1,452 | 55,879 | 58,405 | -2,526 |  |
| 80-84 | 46,747 | 46,942 | -195 | 18,078 | 18,106 | -28 | 28,669 | 28,836 | -167 |  |
| ≥85 | 14,833 | 15,076 | -243 | 5,435 | 5,324 | 111 | 9,398 | 9,752 | -354 |  |
| Total | 503,330 | 495,862 | 7,468 | 208,681 | 205,987 | 2,694 | 294,649 | 289,875 | 4,774 |  |
| Excess^b^ |  |  | 16,424 |  |  | 5,029 |  |  | 11,735 |  |
| ^a^Expected number of cataract surgery was calculated on the assumption that the age group- and gender-specific cataract surgery rate in 2016 was maintained by in subsequent years.  ^b^Excess number of cataract surgeries was calculated by adding up only those of exceeding the expected number of surgeries by age group. | | | | | | | | | | |

|  | 2018 | | | | | | | | |
| --- | --- | --- | --- | --- | --- | --- | --- | --- | --- |
|  | Total | | | Male | | | Female | | |
| Age group | Observed | Expected^a^ | Difference | Observed | Expected | Difference | Observed | Expected | Difference |
| <30 | 1,035 | 1,005 | 30 | 626 | 624 | 2 | 409 | 381 | 28 |
| 30-34 | 571 | 601 | -30 | 349 | 389 | -40 | 222 | 212 | 10 |
| 35-39 | 1,659 | 1,540 | 119 | 1,133 | 995 | 138 | 526 | 545 | -19 |
| 40-44 | 4,906 | 4,373 | 533 | 2,777 | 2,723 | 54 | 2,129 | 1,650 | 479 |
| 45-49 | 19,110 | 13,340 | 5,770 | 8,568 | 7,372 | 1,196 | 10,542 | 5,968 | 4,574 |
| 50-54 | 36,611 | 24,935 | 11,676 | 14,863 | 12,043 | 2,820 | 21,748 | 12,892 | 8,856 |
| 55-59 | 57,099 | 42,897 | 14,202 | 23,510 | 19,522 | 3,988 | 33,589 | 23,375 | 10,214 |
| 60-64 | 73,058 | 66,727 | 6,331 | 30,280 | 28,404 | 1,876 | 42,778 | 38,323 | 4,455 |
| 65-69 | 89,704 | 89,245 | 459 | 36,959 | 36,224 | 735 | 52,745 | 53,021 | -276 |
| 70-74 | 98,082 | 104,637 | -6,555 | 39,846 | 41,761 | -1,915 | 58,236 | 62,876 | -4,640 |
| 75-79 | 97,415 | 98,134 | -719 | 39,277 | 38,873 | 404 | 58,138 | 59,261 | -1,123 |
| 80-84 | 47,966 | 50,102 | -2,136 | 19,310 | 19,639 | -329 | 28,656 | 30,463 | -1,807 |
| ≥85 | 15,370 | 16,284 | -914 | 5,718 | 5,851 | -133 | 9,652 | 10,433 | -781 |
| Total | 542,586 | 513,820 | 28,766 | 223,216 | 214,420 | 8,796 | 319,370 | 299,400 | 19,970 |
| Excess^b^ |  |  | 39,120 |  |  | 11,213 |  |  | 28,616 |

Supplementary material 4. Observed and expected number of cataract surgery according to sex by 5-year intervals (cont.)

| ^a^Expected number of cataract surgery was calculated on the assumption that the age group- and gender-specific cataract surgery rate in 2016 was maintained by in subsequent years.  ^b^Excess number of cataract surgeries was calculated by adding up only those of exceeding the expected number of surgeries by age group. |
| --- |

Supplementary material 4. Observed and expected number of cataract surgery according to sex by 5-year intervals (cont.)

|  | 2019 | | | | | | | | |  |
| --- | --- | --- | --- | --- | --- | --- | --- | --- | --- | --- |
|  | Total | | | Male | | | Female | | |  |
| Age group | Observed | Expected^a^ | Difference | Observed | Expected | Difference | Observed | Expected | Difference |  |
| <30 | 1,012 | 985 | 27 | 641 | 611 | 30 | 371 | 374 | -3 |  |
| 30-34 | 664 | 596 | 68 | 403 | 387 | 16 | 261 | 209 | 52 |  |
| 35-39 | 1,695 | 1,478 | 217 | 1,092 | 957 | 135 | 603 | 521 | 82 |  |
| 40-44 | 5,190 | 4,332 | 858 | 2,872 | 2,691 | 181 | 2,318 | 1,641 | 677 |  |
| 45-49 | 22,832 | 13,134 | 9,698 | 9,625 | 7,270 | 2,355 | 13,207 | 5,864 | 7,343 |  |
| 50-54 | 47,083 | 25,415 | 21,668 | 18,098 | 12,249 | 5,849 | 28,985 | 13,166 | 15,819 |  |
| 55-59 | 70,337 | 42,584 | 27,753 | 27,739 | 19,436 | 8,303 | 42,598 | 23,148 | 19,450 |  |
| 60-64 | 91,380 | 70,927 | 20,453 | 36,524 | 30,137 | 6,387 | 54,856 | 40,790 | 14,066 |  |
| 65-69 | 103,314 | 94,338 | 8,976 | 41,893 | 38,472 | 3,421 | 61,421 | 55,866 | 5,555 |  |
| 70-74 | 114,382 | 109,682 | 4,700 | 47,244 | 44,129 | 3,115 | 67,138 | 65,553 | 1,585 |  |
| 75-79 | 105,741 | 98,451 | 7,290 | 42,922 | 39,338 | 3,584 | 62,819 | 59,113 | 3,706 |  |
| 80-84 | 55,363 | 53,561 | 1,802 | 22,177 | 21,351 | 826 | 33,186 | 32,210 | 976 |  |
| ≥85 | 17,635 | 17,912 | -277 | 6,804 | 6,560 | 244 | 10,831 | 11,352 | -521 |  |
| Total | 636,628 | 533,395 | 103,233 | 258,034 | 223,588 | 34,446 | 378,594 | 309,807 | 68,787 |  |
| Excess^b^ |  |  | 103,510 |  |  | 34,446 |  |  | 69,311 |  |
| ^a^Expected number of cataract surgery was calculated on the assumption that the age group- and gender-specific cataract surgery rate in 2016 was maintained by in subsequent years.  ^b^Excess number of cataract surgeries was calculated by adding up only those of exceeding the expected number of surgeries by age group. | | | | | | | | | | |

Supplementary material 4. Observed and expected number of cataract surgery according to sex by 5-year intervals (cont.)

|  | 2020 | | | | | | | | |  |
| --- | --- | --- | --- | --- | --- | --- | --- | --- | --- | --- |
|  | Total | | | Male | | | Female | | |  |
| Age group | Observed | Expected^a^ | Difference | Observed | Expected | Difference | Observed | Expected | Difference |  |
| <30 | 857 | 962 | -105 | 529 | 596 | -67 | 328 | 366 | -38 |  |
| 30-34 | 507 | 594 | -87 | 318 | 387 | -69 | 189 | 207 | -18 |  |
| 35-39 | 1,600 | 1,408 | 192 | 986 | 912 | 74 | 614 | 496 | 118 |  |
| 40-44 | 5,542 | 4,383 | 1,159 | 2,897 | 2,727 | 170 | 2,645 | 1,656 | 989 |  |
| 45-49 | 27,239 | 12,744 | 14,495 | 10,931 | 7,040 | 3,891 | 16,308 | 5,704 | 10,604 |  |
| 50-54 | 61,148 | 25,757 | 35,391 | 21,908 | 12,438 | 9,470 | 39,240 | 13,319 | 25,921 |  |
| 55-59 | 85,222 | 41,773 | 43,449 | 31,221 | 19,094 | 12,127 | 54,001 | 22,679 | 31,322 |  |
| 60-64 | 105,574 | 74,400 | 31,174 | 40,709 | 31,681 | 9,028 | 64,865 | 42,719 | 22,146 |  |
| 65-69 | 107,018 | 103,567 | 3,451 | 43,392 | 42,159 | 1,233 | 63,626 | 61,408 | 2,218 |  |
| 70-74 | 106,692 | 115,631 | -8,939 | 44,606 | 46,657 | -2,051 | 62,086 | 68,974 | -6,888 |  |
| 75-79 | 89,039 | 98,081 | -9,042 | 36,690 | 39,605 | -2,915 | 52,349 | 58,476 | -6,127 |  |
| 80-84 | 45,024 | 55,583 | -10,559 | 18,641 | 22,469 | -3,828 | 26,383 | 33,114 | -6,731 |  |
| ≥85 | 14,893 | 19,745 | -4,852 | 6,038 | 7,371 | -1,333 | 8,855 | 12,374 | -3,519 |  |
| Total | 650,355 | 554,628 | 95,727 | 258,866 | 233,136 | 25,730 | 391,489 | 321,492 | 69,997 |  |
| Excess^b^ |  |  | 129,311 |  |  | 35,993 |  |  | 93,318 |  |
| ^a^Expected number of cataract surgery was calculated on the assumption that the age group- and gender-specific cataract surgery rate in 2016 was maintained by in subsequent years.  ^b^Excess number of cataract surgeries was calculated by adding up only those of exceeding the expected number of surgeries by age group. | | | | | | | | | | |
