# Supplementary material for: An epidemic of cataract surgery in Korea: the effects of private health insurance on the National Health Insurance Service
Source: Epidemiol Health. 2024 Jan 6;46:e2024015. doi: 10.4178/epih.e2024015 (PMC11099570; doi:10.4178/epih.e2024015)
Supplement: Supplementary Material 5. — Types of intraocular lens for cataract surgery in 2020 [file epih-46-e2024015-Supplementary-5.docx]

Supplementary material 5. Types of intraocular lens for cataract surgery in 2020

|  | Total, N (%) | Use of non-NHIS-covered IOLs^b^, N (%) | Use of NHIS-covered IOLs^b^, N (%) | p-value |
| --- | --- | --- | --- | --- |
| Number of Cataract Surgery^a^ (%) | 619,771 (100.0) | 237,411 (38.3) | 382,360 (61.7) |  |
| Age - mean, SD | 65.84 ± 10.32 | 58.38 ± 7.88 | 70.47 ± 8.84 | <0.001 |
| Age distribution - no. (%) |  |  |  | <0.001 |
| <30 | 769 (0.1) | 427 (55.5) | 342 (44.5) |  |
| 30-34 | 454 (0.1) | 252 (55.5) | 202 (44.5) |  |
| 35-39 | 1,443 (0.2) | 854 (59.2) | 589 (40.8) |  |
| 40-44 | 5,266 (0.9) | 3,734 (70.9) | 1,532 (29.1) |  |
| 45-49 | 26,490 (4.3) | 22,018 (83.1) | 4,472 (16.9) |  |
| 50-54 | 59,658 (9.6) | 49,445 (82.9) | 10,213 (17.1) |  |
| 55-59 | 82,425 (13.3) | 59,535 (72.2) | 22,890 (27.8) |  |
| 60-64 | 100,906 (16.3) | 52,674 (52.2) | 48,232 (47.8) |  |
| 65-69 | 101,382 (16.4) | 29,592 (29.2) | 71,790 (70.8) |  |
| 70-74 | 100,747 (16.3) | 11,917( 11.8) | 88,830 (88.2) |  |
| 75-79 | 84,154 (13.6) | 4,759 (5.7) | 79,395 (94.3) |  |
| 80-84 | 42,294 (6.8) | 1,739 (4.1) | 40,555 (95.9) |  |
| ≥85 | 13,783 (2.2) | 465 (3.4) | 13,318 (96.6) |  |
| <65 | 277,411 (44.8) | 188,939 (68.1) | 88,472 (31.9) | <0.001 |
| ≥65 | 342,360 (55.2) | 48,472 (14.2) | 293,888 (85.8) |  |
| Sex - no. (%) |  |  |  | <0.001 |
| Male | 244,854 (39.5) | 78,715 (32.1) | 166,139 (67.9) |  |
| Female | 374,917 (60.5) | 158,696 (42.3) | 216,221 (57.7) |  |
| Place of Surgery - no. (%) |  |  |  | <0.001 |
| Tertiary hospital | 30,756 (5.0) | 3,477(11.3) | 27,279 (88.7) |  |
| General hospital | 15,616 (2.5) | 1,269 (8.1) | 14,347 (91.9) |  |
| Hospital | 47,224 (7.6) | 17,624 (37.3) | 29,600 (62.7) |  |
| Clinic | 526,175 (84.9) | 215,041 (40.9) | 311,134 (59.1) |  |
| S.D, Standard Deviation; NHIS, National Health Insurance Service; IOLs, intraocular lens  ^a^Only for cataract surgeries reimbursed by DRG payment system  ^b^The percent was calculated are calculated as the horizontal sum was 100. | | | | |
