# Supplementary material for: An epidemic of cataract surgery in Korea: the effects of private health insurance on the National Health Insurance Service
Source: Epidemiol Health. 2024 Jan 6;46:e2024015. doi: 10.4178/epih.e2024015 (PMC11099570; doi:10.4178/epih.e2024015)
Supplement: Supplementary Material 6. — Types of intraocular lens for cataract surgery in 2020 according to sex and age [file epih-46-e2024015-Supplementary-6.docx]

Supplementary material 6. Types of intraocular lens for cataract surgery in 2020 according to sex and age

|  | Male | | | | | | Female | | | | | |
| --- | --- | --- | --- | --- | --- | --- | --- | --- | --- | --- | --- | --- |
| Age group | Total, N (%) | | No use of NHI-covered IOLs, N (%) | | Use of NHI-covered IOLs , N (%) | | Total, N (%) | | No use of NHI-covered IOLs, N (%) | | Use of NHI-covered IOLs, N (%) | |
| <30 | 460 | (0.2) | 267 | (58.0) | 193 | (42.0) | 309 | (0.1) | 160 | (51.8) | 149 | (48.2) |
| 30-34 | 282 | (0.1) | 152 | (53.9) | 130 | (46.1) | 172 | (0.1) | 100 | (58.1) | 72 | (41.9) |
| 35-39 | 883 | (0.4) | 517 | (58.6) | 366 | (41.5) | 560 | (0.2) | 337 | (60.2) | 223 | (39.8) |
| 40-44 | 2,707 | (1.1) | 1,648 | (60.9) | 1,059 | (39.1) | 2,559 | (0.7) | 2,086 | (81.5) | 473 | (18.5) |
| 45-49 | 10,437 | (4.3) | 7,505 | (71.9) | 2,932 | (28.1) | 16,053 | (4.3) | 14,513 | (90.4) | 1,540 | (9.6) |
| 50-54 | 21,087 | (8.6) | 15,087 | (71.6) | 6,000 | (28.5) | 38,571 | (10.3) | 34,358 | (89.1) | 4,213 | (10.9) |
| 55-59 | 29,831 | (12.2) | 18,365 | (61.6) | 11,466 | (38.4) | 52,594 | (14.0) | 41,170 | (78.3) | 11,424 | (21.7) |
| 60-64 | 38,667 | (15.8) | 17,065 | (44.1) | 21,602 | (55.9) | 62,239 | (16.6) | 35,609 | (57.2) | 26,630 | (42.8) |
| 65-69 | 40,965 | (16.7) | 10,496 | (25.6) | 30,469 | (74.4) | 60,417 | (16.1) | 19,096 | (31.6) | 41,321 | (68.4) |
| 70-74 | 41,917 | (17.1) | 4,779 | (11.4) | 37,138 | (88.6) | 58,830 | (15.7) | 7,138 | (12.1) | 51,692 | (87.9) |
| 75-79 | 34,556 | (14.1) | 1,927 | (5.6) | 32,629 | (94.4) | 49,598 | (13.2) | 2,832 | (5.7) | 46,766 | (94.3) |
| 80-84 | 17,532 | (7.2) | 716 | (4.1) | 16,816 | (95.9) | 24,762 | (6.6) | 1,023 | (4.1) | 23,739 | (95.9) |
| ≥85 | 5,530 | (2.3) | 191 | (3.5) | 5,339 | (96.6) | 8,253 | (2.2) | 274 | (3.3) | 7,979 | (96.7) |
| Total |  |  | 78,715 | (32.2) | 166,139 | (67.9) |  |  | 158,696 | (42.3) | 216,221 | (57.7) |
